# Homology modeling and virtual screening approaches to identify potent inhibitors of VEB-1 β-lactamase

**DOI:** 10.1186/1742-4682-10-22

**Published:** 2013-04-02

**Authors:** Abdelmonaem Messaoudi, Hatem Belguith, Jeannette Ben Hamida

**Affiliations:** 1 Unité de Protéomie Fonctionnelle and Biopréservation Alimentaire, Institut Supérieur des Sciences Biologiques Appliquées de Tunis, Université Tunis El Manar, 09, Rue Docteur Zouheïr Safi - 1006, Tunis, Tunisia

**Keywords:** VEB-1 β-lactamase, Homology modeling, Virtual screening, Docking, Inhibitor

## Abstract

**Background:**

*bla*_VEB-1_ is an integron-located extended-spectrum β-lactamase gene initially detected in *Escherichia coli* and *Pseudomonas aeruginosa* strains from south-east Asia. Several recent studies have reported that VEB-1-positive strains are highly resistant to ceftazidime, cefotaxime and aztreonam antibiotics. One strategy to overcome resistance involves administering antibiotics together with β-lactamase inhibitors during the treatment of infectious diseases. During this study, four VEB-1 β-lactamase inhibitors were identified using computer-aided drug design.

**Methods:**

The SWISS-MODEL tool was utilized to generate three dimensional structures of VEB-1 β-lactamase, and the 3D model VEB-1 was verified using PROCHECK, ERRAT and VERIFY 3D programs. Virtual screening was performed by docking inhibitors obtained from the ZINC Database to the active site of the VEB-1 protein using AutoDock Vina software.

**Results and conclusion:**

Homology modeling studies were performed to obtain a three-dimensional structure of VEB-1 β-lactamase. The generated model was validated, and virtual screening of a large chemical ligand library with docking simulations was performed using AutoDock software with the ZINC database. On the basis of the dock-score, four molecules were subjected to ADME/TOX analysis, with ZINC4085364 emerging as the most potent inhibitor of the VEB-1 β-lactamase.

## Background

*bla*_VEB-1_ was identified in 1996 from an *Escherichia coli* strain isolated from a Vietnamese patient. Subsequent analysis demonstrated that *bla*_VEB-1_ is both plasmid- and integron-located [1,2]. Among the different Ambler class A expanded-spectrum β-lactamase (ESBL) genes, *bla*_VEB-1_ is considered to be “emerging”; it has been detected in several Gram-negative organisms including *Enterobacteriaceae* and *Pseudomonas aeruginosa*[3,4], and in multiple countries including France, Spain, Algeria, Turkey, Canada, Korea, Thailand and Tunisia [5-10]. Furthermore, *P*. *aeruginosa* isolates producing the VEB-1a variant, which differs from VEB-1 by only a single amino acid located in the leader peptide of the pre-mature protein, have been identified in Kuwait and India [11,12]. VEB-1 has high amino-acid identity to PER-1 and PER-2 (38%) EBSLs, and confers high-level resistance to ceftazidime, cefotaxime and aztreonam [13]. The *bla*_VEB-1_ gene was characterized in an unusual genetic environment in *P*. *aeruginosa* isolates from India and Bangladesh, and in *P*. *stuartii* from Algeria. Rather than having a typical class 1 integron structure, in these isolates *bla*_VEB-1_ is flanked by identical 135-bp sequences, termed repeated elements (Res), which are bracketed by two truncated 3^′^-conserved class 1 integron sequences in direct repeat [14].

There are currently no clinically useful inhibitors of VEB-1 β-lactamase. However, several studies have been undertaken using a variety of experimental inhibitors against other Ambler class A ESBLs. Clavulanic acid, the first β-lactamase inhibitor introduced into clinical medicine, was isolated from *Streptomyces clavuligerus* during the 1970s [15]. Clavulanate (the salt form of the acid in solution) presented with little antimicrobial activity in isolation, but when combined with amoxicillin, it significantly lowered amoxicillin MICs against *Staphylococcus aureus*, *Klebsiella pneumoniae*, *Proteus mirabilis* and *E. coli*[16]. Sulbactam and tazobactam are penicillinate sulfones developed as synthetic compounds in 1978 and 1980, respectively [17,18]. Class A β-lactamase is inhibited to comparable levels by moxalactam, imipenem and cefoxitin.

The crystal structure of VEB-1 β-lactamase has not been described. Determining the three-dimensional (3D) structure of this molecule would assist in the discovery of more potent inhibitors, particularly in the application of structure-based virtual screening to identify lead compounds. To this end, a homology model of the 3D structure of VEB-1 protein was produced and a computational docking process was used to identify a series of potent inhibitors from the ZINC Database to allow VEB-1 to be compared with other Class A β-lactamase complexes.

## Methods

### Template identification and protein homology modeling

**Figure 1:**
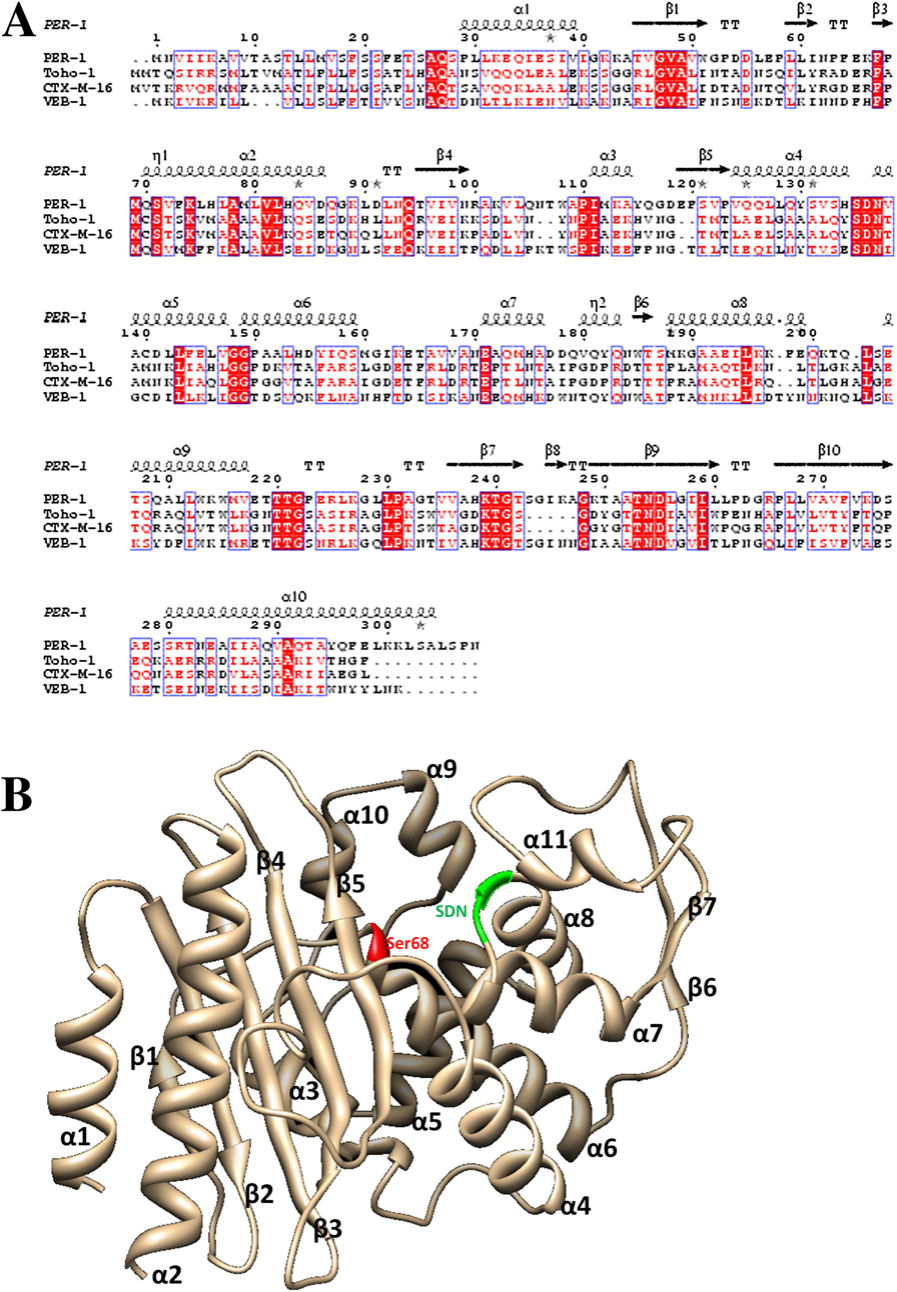
**Overall structure of VEB-1 and its sequence alignment with its homologue proteins. A**. Sequence alignment of VEB-1 with PER-1, Toho-1 and CTX-M-16. The second structure assignment of PER-1 is labeled on the top of the sequences. **B**. Cartoon representation of the overall structure of VEB-1 is in light orange color. The serine active-site is colored in red, the SDN motif in green.

Searching the RCSB Protein Data Bank (http://www.rcsb.org/) confirmed that the tertiary structure of VEB-1 β-lactamase was not publicly available. The complete *E. coli* VEB-1 β-lactamase protein sequence, which consists of 299 amino acids and has a calculated molecular weight of 33.7 kDa, was retrieved from the UniProtKB database (http://www.uniprot.org/) (accession number Q7BVU7). BLASTP [19] was used to identify homologs in the RCSB Protein Databank [20]. Accordingly, the crystal structure of PER-1 β-lactamase from *P. aeruginosa* (PDB ID: 1E25), which has 40% sequence identity to VEB-1, was selected as the template [21]. To analyze sequence conservation, the VEB-1, PER-1, CTX-M and Toho-1 sequences were aligned. Gaps were inserted into the sequences to discover an optimal alignment, as presented in Figure 1A. The 3D structure of VEB-1 was modeled using the SWISS-MODEL tool [22] in the ExPASy Bioinformatics resource portal [23], and viewed using Swiss PDB Viewer v 4.0.1 software [24].

### Model optimization and evaluation

Protein models generated using homology modeling frequently produce unfavorable bond lengths, bond angles, torsion angles and contacts. Therefore, it was essential to minimize the energy to regularize local bond and angle geometry, and to relax close contacts in the geometric chain. Each model of VEB-1 was optimized using the variable target function method (VTFM) with conjugate gradients (CG), followed by further refinement using molecular dynamics (MD) with a simulated annealing (SA) method in Modeller [25]. Energy minimization was performed to minimize stearic collisions and strains without significantly altering the overall structure. Energy computations and minimization were carried out using the GROMOS96 force field [26] and implementing Swiss-PdbViewer. After optimization the 3D model of VEB-1 was verified using the PROCHECK [27], ERRAT [28] and VERIFY 3D [29] programs available from the Structural Analysis and Verification Server (SAVES) (http://nihserver.mbi.ucla.edu/SAVES). PROCHECK was used to assess the stereochemical quality of the protein structure, while the Verify3D program analyzed the compatibility of an atomic model (3D) with its own amino acid sequence (1D) to assess the 3D protein structure.

### Screening of compounds from the ZINC Database

Ligand-based virtual screening experiments are important during the early stages of drug discovery, as they can screen compound databases using the active sites of proteins with known 3D structure. The ZINC Database [30] is free to use and contains commercially available chemical compounds prepared for virtual screening. It contains more than 21 million compounds in ready-to-dock, 3D formats that can be purchased. During this work the ZINC Database was screened for structurally similar inhibitors of VEB-1 β-lactamases. The compounds identified included clavulanic acid, sulbactam, tazobactam, imipenem, cefoxitin and moxalactam. Furthermore, this study identified 950 compounds that were structurally similar to available Amber class A β-lactamase inhibitors during screening.

### Structure-based virtual screening using molecular docking

Virtual screening uses computational methods to identify molecules that are biologically active against a specific protein target [31]. Two types of methodologies can be used during virtual screening: those that search for similarity to validated ligands, and molecular docking methods that require structural information about the target. During this study the first method, which is also known as ligand-based virtual screening, was utilized. Analogs with a minimum of 70% similarity to the known β-lactamase inhibitors (clavulanic acid, sulbactam, tazobactam, imipenem, cefoxitin and moxalactam) were selected from the ZINC database. To remove structural redundancies from the chemical library, structurally similar compounds with a Tanimoto coefficient larger than 0.8 were clustered into a single representative molecule. As a consequence, a docking library consisting of 950 compounds was obtained and downloaded in mol2 format.

Virtual screening was performed by docking the inhibitors obtained from the ZINC database to the active site of the VEB-1 protein using AutoDock Vina software (version 1.0) [32]. This docking allowed a population of possible conformations and orientations for the ligand at the binding site to be obtained. Using the Autodock Tools software [33], polar hydrogen atoms were added to VEB-1 protein, and its non-polar hydrogen atoms were merged. The protein receptor (VEB-1) and inhibitors were converted from PDB format to PDBQT format. All bonds within ligands were set to allow rotation. In the configuration file of the Autodock Vina software, a grid box with dimensions of 20 × 20 × 20 points was used around the active site to cover the entire enzyme binding site and allow ligands to move freely.

The docking simulation of each compound was conducted using an improved empirical AutoDock scoring function, in which a new solvation model for organic molecules was introduced. This modified scoring function can be expressed as follows:

(1) $$\begin{array}{l} \Delta G_{\mathrm{bind}}^{\mathrm{aq}}=W_{\mathrm{vdW}}\sum_{i=1}\sum_{j>i}\left(\frac{A_{\mathrm{ij}}}{r_{\mathrm{ij}}^{12}}-\frac{B_{\mathrm{ij}}}{r_{\mathrm{ij}}^{6}}\right)+W_{\mathrm{hbond}}\sum_{i=1}\sum_{j>i}E\left(t\right)\left(\frac{A_{\mathrm{ij}}}{r_{\mathrm{ij}}^{12}}-\frac{B_{\mathrm{ij}}}{r_{\mathrm{ij}}^{6}}\right)\\ +W_{\mathrm{elec}}\sum_{i=1}\sum_{j>i}\frac{q_{i}q_{i}}{\varepsilon \left(\mathrm{rij}\right)\mathrm{rij}}+W_{\mathrm{tor}}N_{\mathrm{tor}}+\;W_{\mathrm{sol}}\sum_{i=1}S_{i}\left(\mathrm{Oc}c_{i}^{\max}-\sum_{i>i}V_{j}e^{-\frac{r_{\mathrm{ij}}^{2}}{2\sigma^{2}}}\right) \end{array}$$

where *WvdW, Whbond, Welec, Wtor* and *Wso*l are the weighting factors of van der Waals, hydrogen bond, electrostatic interactions, torsional term and desolvation energy of the inhibitors, respectively. *rij* represents the interatomic distance, and *Aij*, *Bij*, *Cij* and *Dij* are related to the depths of the potential energy well and the equilibrium separations between the two atoms. The hydrogen bond term has an additional weighting factor, *E(t)*, representing the angle-dependent directionality. The cubic equation approach was applied to obtain the dielectric constant required for computing the interatomic electrostatic interactions between VEB-1 and a ligand molecule [34]. In the entropic term, *Ntor* is the number of sp^3^ bonds in the ligand. In the desolvation term, *Si* and *Vi* are the solvation parameter and the fragmental volume of atom *i*, [35] respectively, while *Occi*^*max*^ is the maximum atomic occupancy. In the calculation of the molecular solvation free energy term in Eq. (1), the atomic parameters developed by Kang et al. [36] were used, as only carbon atoms were available. This modification of the solvation free energy term is expected to increase the accuracy of virtual screening, as underestimation of ligand solvation can lead to overestimation of the binding affinity of a ligand with several polar atoms [37].

The best conformation with the lowest docked energy was chosen from the docking search. The interactions of complex VEB-1 protein-ligand conformations including hydrogen bonds and bond lengths were analyzed using Swiss-PdbViewer v4.0 [38], Pymol software [39], UCSF Chimera [40] and Accelrys DS Visualizer software [41]. The commercially available software toxtree (developed by Idea consult Ltd., Sofia, Bulgaria) was used for computer-based estimation of chemical toxicity [42].

## Results and Discussion

### Protein homology modeling and validation

Multiple sequence alignment of VEB-1 with PER-1, CTX-M-16 and Toho-1 β-lactamases demonstrated that VEB-1 is highly homologous to PER-1 type β-lactamases (38% sequence identity) (Figure 1A). The BLASTP homology search using the *E. coli* VEB-1 β-lactamase sequence against the PDB database confirmed this result (data not shown). In addition, sequence alignment indicated that VEB-1 contains a serine-valine-methionine-lysine tetrad (SXXK) at positions 70–73, including the conserved serine and lysine amino acid residues that are characteristic of β-lactamases with a serine active site [43]. Several other structural elements characteristic of class A β-lactamases were identified including a serine-aspartate-asparagine (SDN) motif at positions 135–137, and lysine-threonine-arginine (KTG) residues at positions 239–242 (Figure 1A.). Accordingly, the crystal structure of PER-1 β-lactamase from *P. aeruginosa* (PDB ID: 1E25) was used as the template during homology modeling.

The modeled enzyme is a monomer, folded into an α/β domain consisting of a seven-stranded β-sheet and 11 α-helices (Figure 1B.). The residues in the Ser135-Asp136-Asn137 (SDN) motif are involved in maintaining the structure of the active site cavity, enzyme stability and stabilization of the enzyme transition state, respectively [44]. Ser135, a conserved amino acid among all class A β-lactamases, is occasionally replaced by a Gly residue. The multiple roles of this residue include anchoring β-lactams to the active site, and stabilizing it, through hydrogen bonding with the C-3/C-4 carboxylates of inhibitors and substrates and facilitating proton transfer to the β-lactam nitrogen during acylation, leading to opening of the β-lactam ring [45].

**Figure 2:**
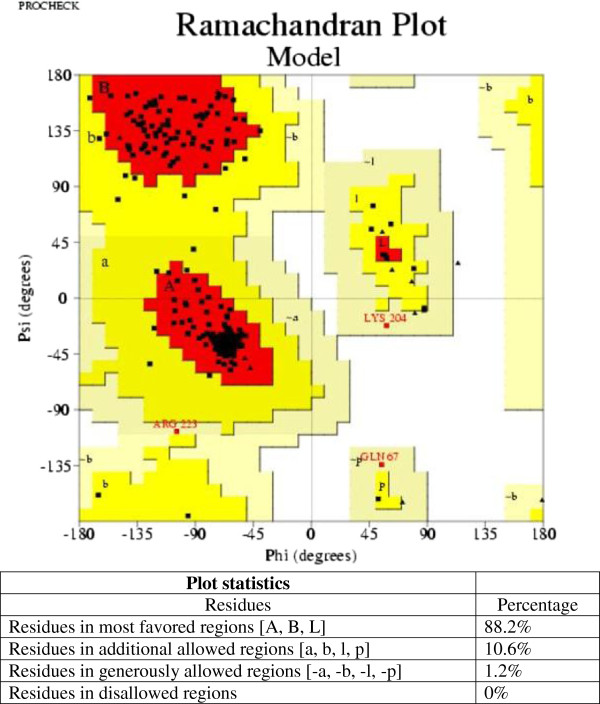
**Ramachandran plot of VEB-1 β-lactamase model from *Escherichia coli *obtained by PROCHECK: 88.2% residues in favorable regions; 10.6% residues in additional allowed regions; 1.2% residues in generously allowed regions; 0% residues in disallowed regions.**

**Figure 3:**
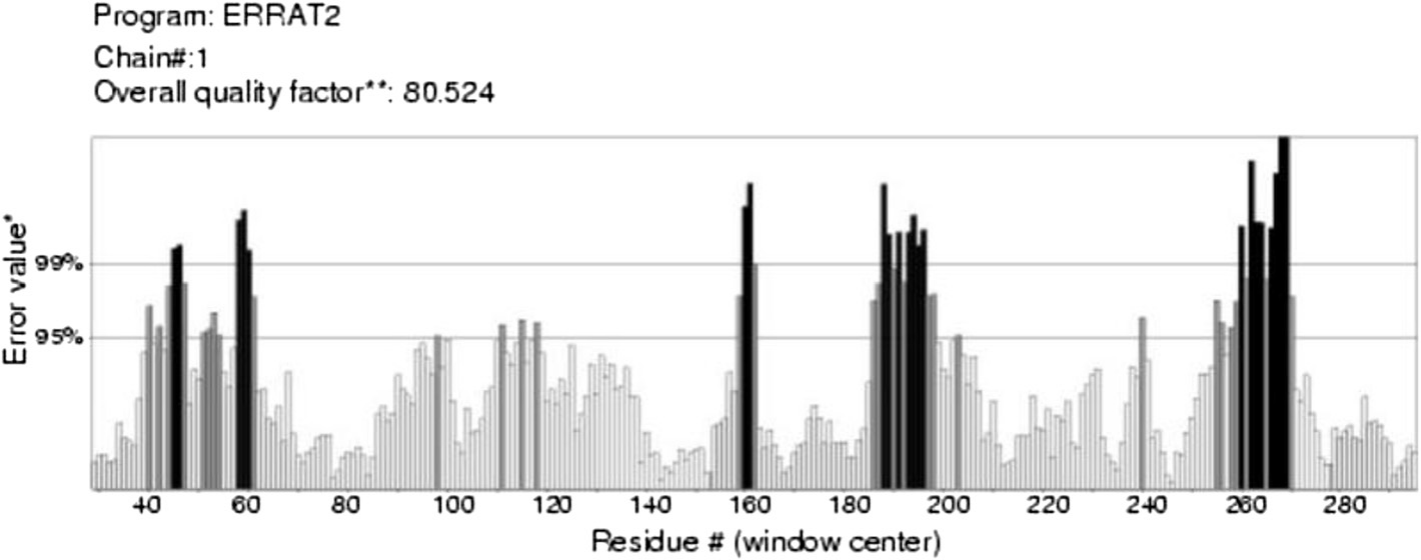
**Errat plot for the VEB-1 β-lactamase model.** Black bars identify the misfolded region located distantly from the active site, gray bars demonstrate the error region between 95% and 99%, and white bars indicate the region with a lower error rate for protein folding.

The quality of the 3D model was evaluated via the Ramachandran plot using PROCHECK software (Figure 2). The Ramachandran plot for the predicted model revealed that 88.2% of residues were in the most favorable region, while 10.6% were in the allowed region, confirming that the predicted model is of good quality. ERRAT is a so-called “overall quality factor” for non-bonded atomic interactions, with higher scores indicating higher quality. The generally accepted range is >50 for a high quality model. For the current 3D model, the overall quality factor predicted by the ERRAT server was 80.524 (Figure 3). The Verify 3D server predicted that 88.77% of the residues in VEB-1 β-lactamase had an average 3D-1D score > 0.2, thereby verifying the model.

### Virtual screening result analysis

**Figure 4:**
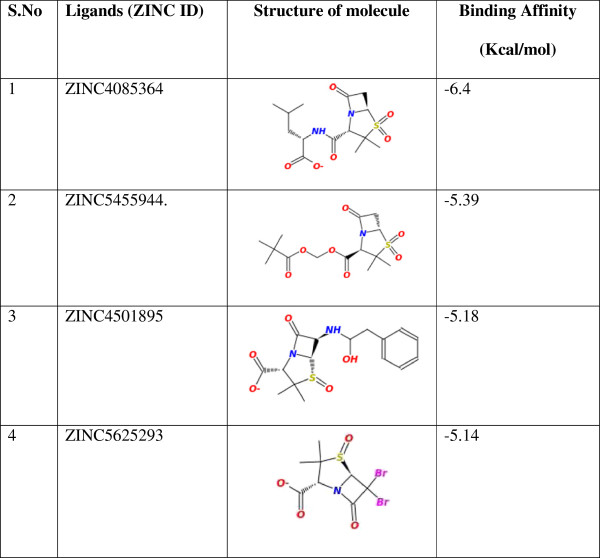
Estimated Free Energy of Binding of the top four ligands.

Following docking simulations, the four most promising inhibitors were selected on the basis of binding affinity (Figure 4). Among the six categories of Amber class A β-lactamase inhibitors considered during the analysis, the optimal interactions with the highest affinity scores were obtained with sulbactam analogs. This finding was contrary to previous results for SHV-1 Amber class A β-lactamase, and sulbactam is a less potent inhibitor than clavulanate [46]. Sulbactam is more potent against class C β-lactamases than clavulanate, whereas its activity against class D enzymes is less potent than against class A β-lactamases. Similarly, sulbactam does not inhibit OXA-type enzymes as efficiently as TEM-1 and other clinically used inhibitors [47].

**Figure 5:**
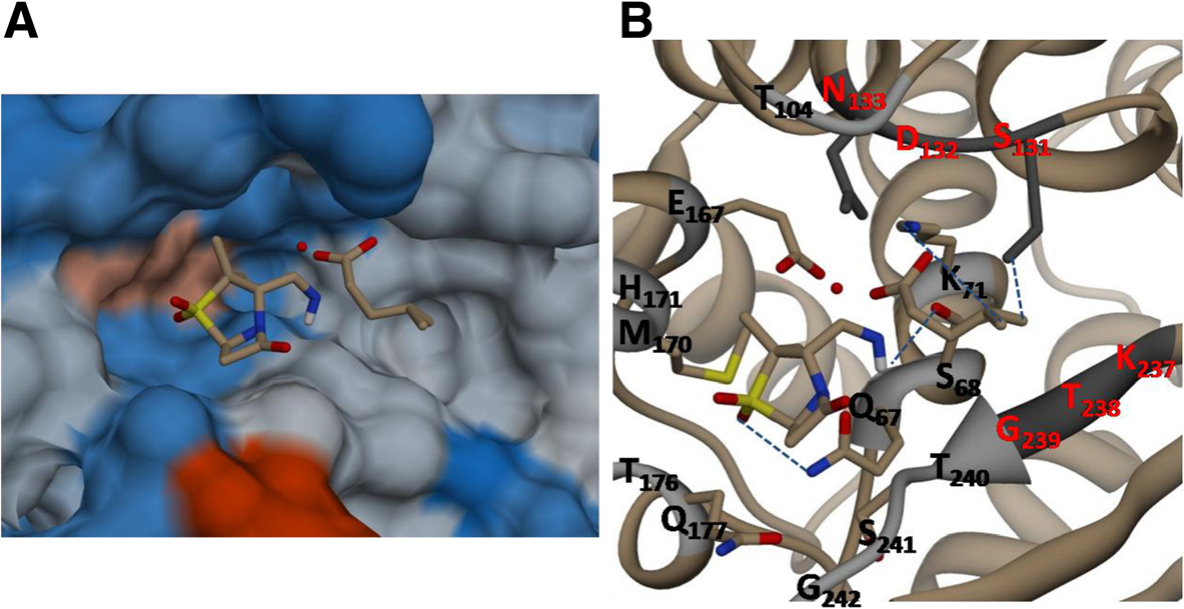
**Binding interactions of inhibitors demonstrating maximum binding affinity (ZINC4085364), and the modeled VEB-1 protein.** The left presents the structural electrostatic surface, and the right the detailed binding interactions. (hydrogen bond interactions are indicated with blue dotted lines).

The best conformation demonstrated that the free energy of binding (ΔGbind, kcal/mol) for the top four inhibitors was good. The negative and low value of ΔGbind (-6.4) indicated strong bonds between VEB-1 and the ZINC4085364 inhibitor, and demonstrated that the inhibitor was in its most favorable conformation. Analysis of the docked complexes demonstrated that the inhibitor was located close to the active site (Ser68), at a distance of 0.6 Å. The complex was stabilized by four hydrogen bonds through residues Ser68, Lys71, Ser131 and Gln67 (Figure 5B). The residue involved in cavity formation is presented in Figure 5A. Interaction analysis revealed that the cavity involved in the binding site has a volume of 186.6 Å^3^ and a surface area of 178.2 Å^2^. Toxtree was used to estimate toxic properties. Finally, four molecules were selected (Table 1).

**Table 1:** Physiochemical properties of the most potent ligand (ZINC4085364) obtained from the docking study

pH range	**Reference (pH 7)**
xlogP	−1.64
Apolar desolvation (kcal/mol)	−7.31
Polar desolvation (kcal/mol)	−66.16
H-bond donors	1
H-bond acceptors	8
Net charge	−1
tPSA (Å^2^)	123
Molecular weight (g/mol)	345.397
Rotatable bonds	5
Popular name	(S)-2-((2S,5R)-3,3-dimethyl-4,4-dioxido-7-oxo-4-thia-1-azabicyclo[3.2.0]heptane-2-carboxamido)-4-methylpentanoic acid
Molecular formula	C14H22N2O6S

## Conclusions

Antibiotic resistance is one of the most serious threats to public health. Development of resistance is assisted by the existence of plasmids, which can be transmitted easily between bacteria. The emergent VEB-1 β-lactamase possesses potent hydrolysis activity towards almost all antibiotics and is a significant threat. Virtual screening is an important tool for exploring biologically relevant chemical spaces, and allows studies focused on small molecule libraries to be performed, using up to millions of compounds. During the present study, structural models of a VEB-1/ZINC4085364 inhibitor complex were obtained using homology modeling and molecular docking methods. At present, there are no effective antibiotics against VEB-1-positive pathogens. An appropriate strategy involves identifying drug candidates from existing antibiotics, such as cephalosporin, on the basis of the 3D model of VEB-1 using structure-based virtual screening. This strategy was used successfully in the discovery of Merck’s HIV protease inhibitor [48]. The molecule identified in the current study as a VEB-1 inhibitor could be exploited for drug design. However, further *in vivo* experimentation is required for complete evaluation.

## Competing interests

The authors declare that they have no competing interests.

## Authors’ contributions

AM carried out all analyses and drafted the manuscript under the guidance of HB and JBH. All authors read and approved the final manuscript.
